# The evolution and future of diabetic kidney disease research: a bibliometric analysis

**DOI:** 10.1186/s12882-021-02369-z

**Published:** 2021-04-29

**Authors:** Yi Wei, Zongpei Jiang

**Affiliations:** Department of Nephrology, The Sixth Affiliated Hospital, Sun Yat-sen University, 26 Yuancun Er Heng Road, Guangzhou, 510655 China

**Keywords:** DKD, RAAS, ACE2, SGLT-2, Bibliometric analysis

## Abstract

**Background:**

Diabetic kidney disease (DKD) is one of the most important complications of diabetic mellitus. It is essential for nephrologists to understand the evolution and development trends of DKD.

**Methods:**

Based on the total cited numbers in the Web of Science Core Collection, which was searched through September 28th, 2020, we performed a bibliometric analysis of the top 100 most cited full-length original articles on the subject of DKD. The timespans, authors, contributions, subcategories, and topics of those 100 articles were analysed. In addition, the evolution of topics in DKD research was investigated.

**Results:**

There were 23,968 items under the subject of DKD in the Web of Science Core Collection. The top 100 cited articles, published from 1999 to 2017, were cited 38,855 times in total. Researchers from the USA contributed the most publications. The number of articles included in ‘Experimental studies (EG)’, ‘Clinical studies (CS)’, ‘Epidemiological studies (ES)’, and ‘Pathological and pathophysiological studies (PP)’ were 65, 26, 7, and 2, respectively. Among the 15 topics, the most popular topic is the renin-angiotensin-aldosterone system (RAAS), occurring in 26 articles, including 6 of the top 10 most cited articles. The evolution of topics reveals that the role of RAAS inhibitor is a continuous hotspot, and sodium-glucose cotransporter 2 (SGLT-2) inhibitor and glucagon-like peptide 1 (GLP-1) agonist are two renoprotective agents which represent novel therapeutic methods in DKD. In addition, the 26 clinical studies among the top 100 most cited articles were highlighted, as they help guide clinical practice to better serve patients.

**Conclusions:**

This bibliometric analysis of the top 100 most cited articles revealed important studies, popular topics, and trends in DKD research to assist researchers in further understanding the subject.

**Supplementary Information:**

The online version contains supplementary material available at 10.1186/s12882-021-02369-z.

## Background

Diabetes mellitus (DM) is a severe global health problem and contributes to increased health care costs. It is estimated that more than 450 million people are affected by this disease, and this number will reach 700 million people by 2045 [1]. Diabetic kidney disease (DKD) is one of the most important complications of DM, and chronic kidney disease occurs in more than 20–40% of DM patients [2]. Thus, overall comprehension of the mechanism and treatment of DKD is essential for nephrologists, especially young researchers.

Recent reviews have helped researchers understand the mechanism, diagnosis, and treatment of DKD, as summarized in Table S1. These studies highlight the vital roles of immunity and inflammation [3–5], oxidative stress (OS) [6–8], haemodynamic and metabolic shifts [9], epigenetic factors [10] and tubule function [11] in the pathogenesis and prognosis of DKD. However, these content-based reviews have 3 limitations. First, the review contents are deep but narrow, which makes it difficult for readers to understand the overall research status of the subject. Second, the articles referenced in the reviews were manually selected. The large workload leads to limited numbers. However, subjective judgement may lead to the loss of information. Authors may miss important research. Even if an article is cited, its importance and influence may be neglected. Third, readers cannot learn the evolution of the subject or evolution of popular topics and thus cannot develop a deeper comprehension of the subject.

Clinical and experimental studies on DKD have developed rapidly. In total, there were 23,968 items in the Web of Science Core Collection on the subject of DKD from 1999 to September 28th, 2020. In a sense, the citation time (CT) represents the influence and significance of the article. Bibliometric analysis is a method utilized in many fields to illustrate the landscape of subjects [12, 13]. Based on CT, bibliometric analysis objectively includes articles of subject on subject in the analysis and reflects the evolution of a subject, trends of popular topics and collaborative relationships among researchers and countries [14–16]. To determine the influential research, the distribution of disparate topics and the evolution of research trends, we performed a bibliometric analysis of the top 100 most cited articles in clinical and experimental studies of DKD.

## Materials and methods

### Data collection and filtration

To acquire literature data representing high-quality research, we retrieved publications on the subject of DKD from the Web of Science Core Collection using the search strategy: TOPIC: (DKD OR (diabetic NEAR/0 nephropathy) OR (diabetic NEAR/0 kidney)). Research results were ranked by CT, which was based on the absolute number of citations for each article through September 28th, 2020. Articles involving the following research objects were defined as publications on the subject of DKD and were manually selected: DKD populations; diabetes populations with proteinuria or kidney disease; tissue, blood, or urine from DKD and diabetes patients with proteinuria or kidney disease; diabetic animals with kidney injury; and renal cell models simulating diabetes injury. Only full-length original articles were included in this study while other types of articles, such as guidelines, reviews, and meta-analyses, were excluded. Finally, only the top 100 most cited full-length original articles written in English on the subject of DKD were included in this study.

### Data analysis and visualization

Elements including the article title, author, address, abstract, keyword, journal, publication year and CT were included in the analysis. The article number was equal to the article rank among the 100 articles. The relevant countries were analysed according to the corresponding authors. If the corresponding authors came from the same country, the article was defined as a single-country publication; otherwise, it was defined as a multi-country publication. Subcategories and topics were manually summarized and counted. The average citation time (ACT) of each article was equal to the CT divided by the number of years the article was cited, and the number of years the article was cited was equal to 2020 plus 1 minus the number of the published year. In addition, the number of citations per article per year was equal to the sum of ACTs of all articles published in the same year divided by the number of articles. The author’s influence score was represented by the sum of the ACT, and one article was scored on only the publication year.

Bibliometric analysis was performed using the Bibliometric R Package [17]. Journal analysis was performed using the module of most relevant sources. Country analysis was performed using the module of the corresponding author’s country. The frequencies of key words included in the word cloud were determined using the module of most frequent words with abstract parameters.

**Fig. 1:**
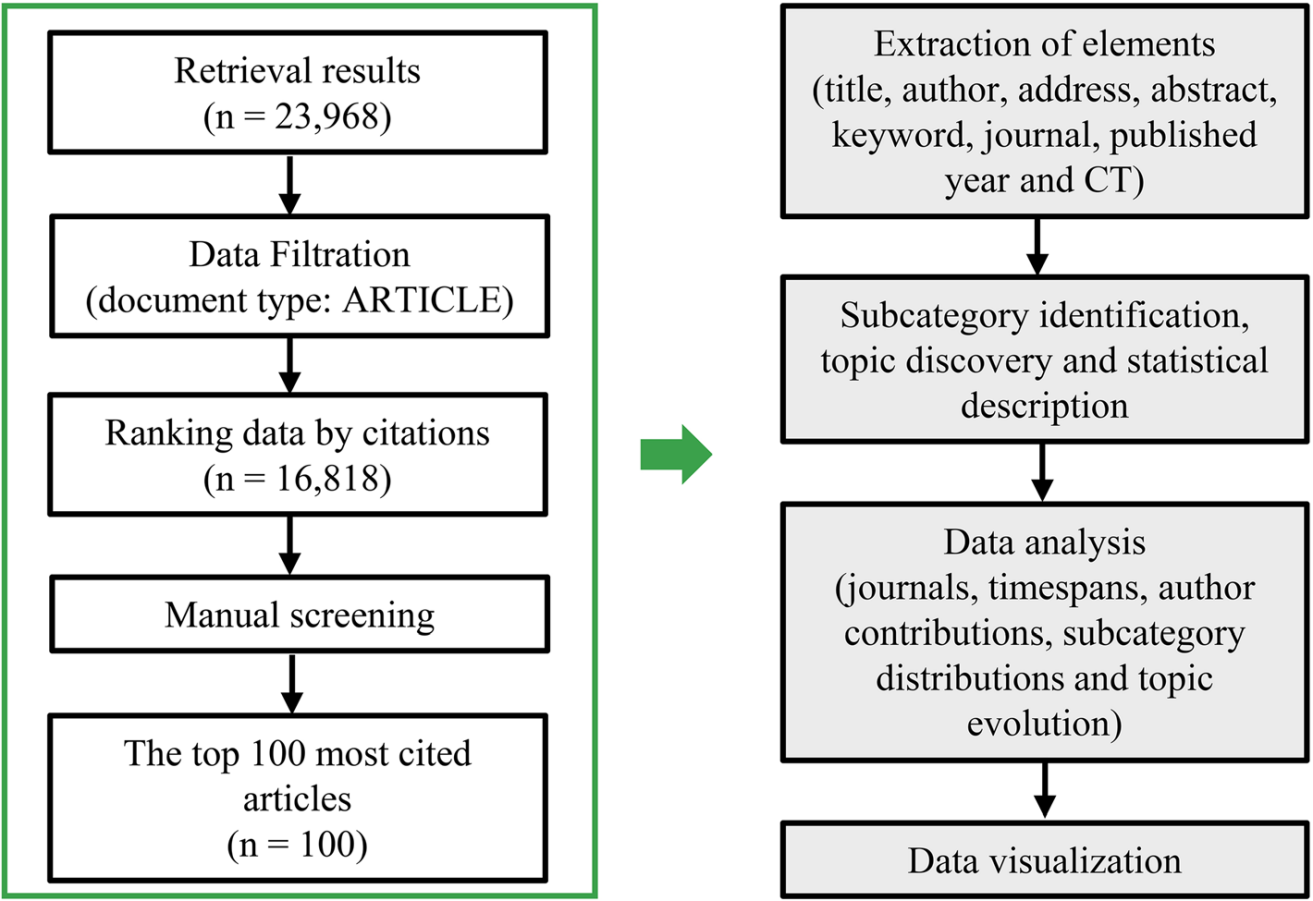
Strategy for data preparation and analysis. We retrieved publications on the subject of DKD from the Web of Science Core Collection and ranked the top 100 most cited articles. After subcategory and topic identification, we performed analyses of the journals, countries, author contributions, distributions and topic evolution

Figures were made by Microsoft PowerPoint, ggplot2 R Package, and ggwordcloud R Package. The process of data preparation and analysis is shown in Fig. 1.

## Results

### Basic information of the top 100 most cited articles

As shown in Table 1, the top 100 most cited articles were published in 26 journals, and 55% of the articles were published in the following 4 journals: Journal of the American Society of Nephrology (21%), Diabetes (16%), Kidney International (10%), and The New England Journal of Medicine (8%).

**Table 1:** List of the relevant scientific journals

Journal	Number of articles
J Am Soc Nephrol	21
Diabetes	16
Kidney Int	10
N Engl J Med	8
J Clin Invest	6
JAMA	4
J Biol Chem	4
Am J Physiol Renal Physiol	3
Hypertension	3
Nat Med	3
Proc Natl Acad Sci U S A	3
Am J Pathol	2
Diabetes Care	2
FASEB J	2
Nephrol Dial Transplant	2
Am J Kidney Dis	1
Am J Nephrol	1
Ann Intern Med	1
Arch Intern Med	1
Biochim Biophys Acta	1
Cell Metab	1
Circulation	1
Diabetes Obes Metab	1
Diabetologia	1
Lancet	1
Nat Cell Biol	1

**Fig. 2:**
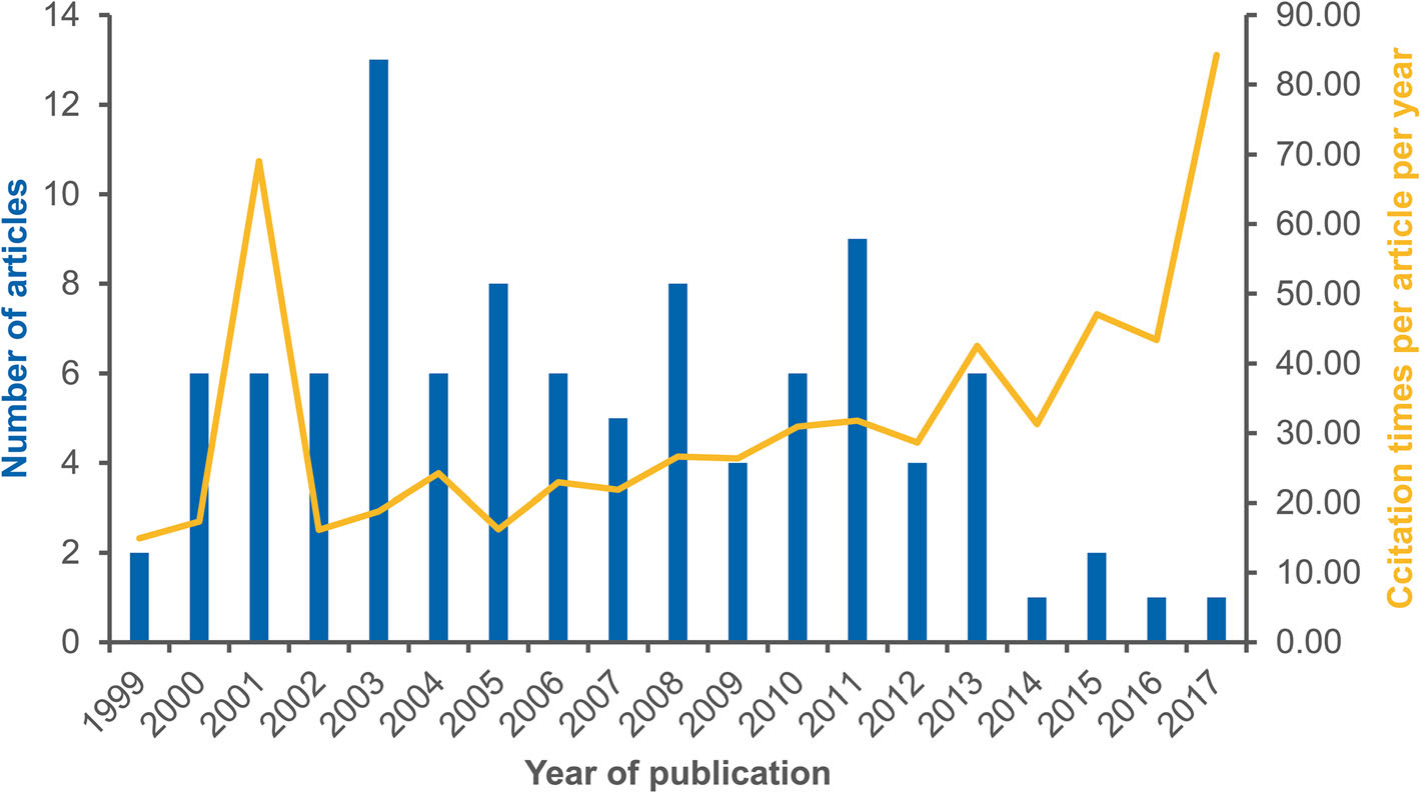
Timespans of the top 100 most cited articles. The blue bars represent the number of publications for each year, and the yellow line represents the number of citations per article per year. The distributions of articles and citation in disparate years are shown in the figure

The 100 articles were published from 1999 to 2017 and were cumulatively cited 38,855 times. The publication time spans are shown in Fig. 2. There were 20 articles published from 1999 to 2002, 38 articles published from 2003 to 2007, 31 articles published from 2008 to 2012 and 11 articles published from 2013 to 2017. In 2003, 13 articles were published, which made 2013 the year with the most publications.

Finally, we analysed the authors, and their affiliations, of the top 100 most cited articles. The authors shown in Figure S1 were the top 10 most relevant authors ranked by the author’s influence score. The dot size represents the score of authors, and the dot colour reflects the number of works published by the author. In Table 2, we found that scientists from the USA contributed the most publications.

**Table 2:** List of the relevant countries analysed according to the corresponding author

Country	Number of articles	Single-county publication	Multiple-country publication
USA	47	24	23
Japan	14	8	6
Australia	11	6	5
Germany	5	1	4
Netherland	5	0	5
Denmark	4	0	4
UK	3	2	1
Canada	2	0	2
Finland	2	0	2
Belgium	1	0	1
Chile	1	0	1
China	1	1	0
India	1	1	0
Ireland	1	0	1
Italy	1	0	1
Korea	1	0	1

### Identification of the subcategories of the top 100 articles

We divided the articles into 4 subcategories according to the research type and content. The most abundant article subcategory was ‘Experimental studies (EG)’, which explained the phenotypic changes and mechanisms in experimental DKD and comprised 65 articles (49.62% of all citations). The second most abundant subcategory was ‘Clinical studies (CS)’, which focused on the clinical biomarkers and treatment strategies for DKD, and in this subcategory, 26 articles contributed to 41.55% of the total citations. Moreover, 7 articles with 2603 total citations were determined to belong to the subcategory ‘Epidemiological studies (ES)’, and 2 articles specifically describing the pathological and pathophysiological characteristics of DKD belonged to the subcategory ‘Pathological and pathophysiological studies (PP)’. In Table 3, we list the article number and percentage of total citations for each subcategory.

**Table 3:** Subcategories of the top 100 most cited articles

Subcategory	Description	Article Number	CT (total)	% total
Experimental studies (EG) *n* = 65	Studies on mechanisms, metabolomics, transcriptome, genetics, potential molecular biomarkers	5, 6, 12, 13, 19–25, 28–31, 33, 34, 37–40, 42, 43, 45, 48, 49, 51, 53–57, 59–61, 63, 65, 67–74, 77–79, 81–85, 87–92, 94–97, 99, 100	19,280	49.62
Clinical studies (CS) *n* = 26	Clinical trials, observational studies	1–3, 7–10, 14, 15, 17, 18, 27, 32, 35, 36, 41, 44, 50, 52, 62, 64, 66, 75, 80, 86, 93	16,144	41.55
Epidemiological studies (ES) *n* = 7	Studies on prevalence, risk factors, outcomes	4, 16, 26, 46, 58, 76, 98	2603	6.70
Pathological and pathophysiological studies (PP) *n* = 2	Studies on pathology and pathophysiology	11, 47	828	2.13

**Fig. 3:**
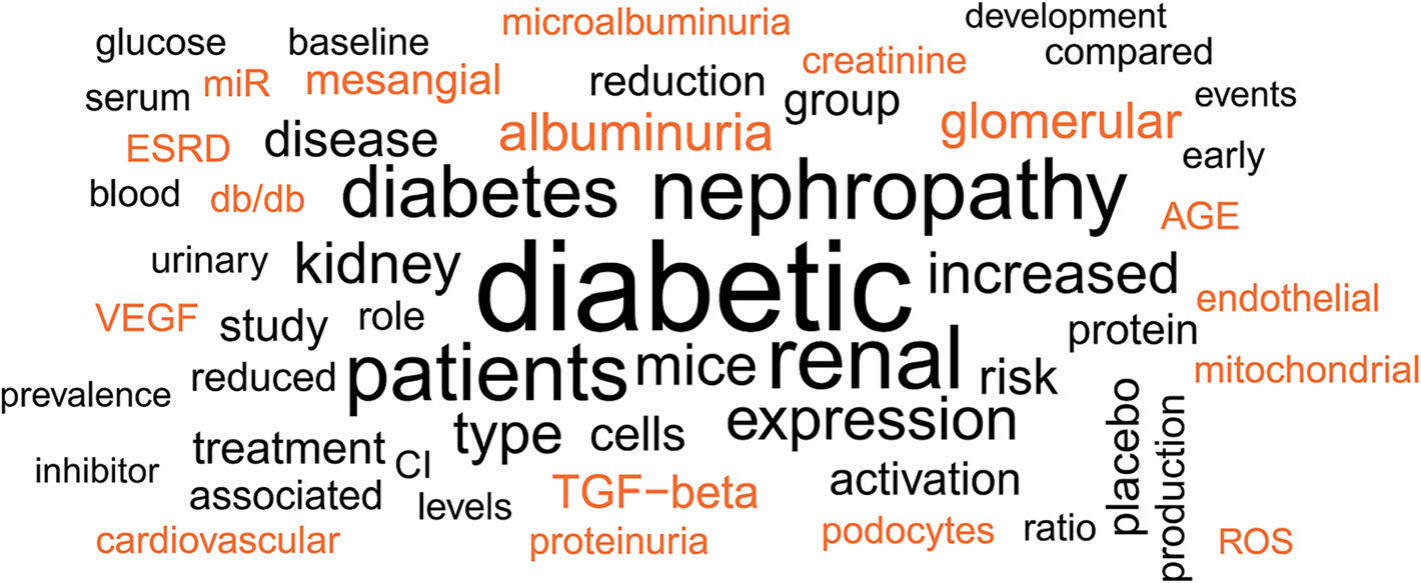
Word cloud of the top 100 most cited articles. Key words were extracted from the abstracts of the top 100 most cited articles to illustrate a word cloud

To learn more about the contents of the 100 articles, we analysed the key words. Figure 3 shows the highly frequent words related to the top 100 most cited articles on DKD. To further determine the hotspots in DKD research, we analysed articles in the subcategories ‘EG’ and ‘CS’ by their topic. In Table 4, we list the top 100 most cited articles and their topics.

**Table 4:** List of the top 100 most cited articles

Number	Year	Author	Title	Journal	CT (ACT)	Subcategory	Topic
1	2001	Brenner BM, et al	Effects of losartan on renal and cardiovascular outcomes in patients with type 2 diabetes and nephropathy	N Engl J Med	4699 (234.95)	CS	RAAS
2	2001	Parving HH, et al	The effect of irbesartan on the development of diabetic nephropathy in patients with type 2 diabetes	N Engl J Med	2229 (111.45)	CS	RAAS
3	2008	Parving HH, et al	Aliskiren combined with losartan in type 2 diabetes and nephropathy	N Engl J Med	769 (59.15)	CS	RAAS
4	2003	Steffes MW, et al	Sustained effect of intensive treatment of type 1 diabetes mellitus on development and progression of diabetic nephropathy - the Epidemiology of Diabetes Interventions and Complications (EDIC) study	JAMA	751 (41.72)	ES	Undefined
5	2006	Susztak K, et al	Glucose-induced reactive oxygen species cause apoptosis of podocytes and podocyte depletion at the onset of diabetic nephropathy	Diabetes	731 (48.73)	MG	OS
6	2000	Ziyadeh FN, et al	Long-term prevention of renal insufficiency excess matrix gene expression and glomerular mesangial matrix expansion by treatment with monoclonal antitransforming growth factor-beta antibody in db/db diabetic mice	Proc Natl Acad Sci U S A	707 (33.67)	MG	TGF-β
7	2004	de Zeeuw D, et al	Proteinuria, a target for renoprotection in patients with type 2 diabetic nephropathy: lessons from RENAAL	Kidney Int	676 (39.76)	CS	RAAS
8	2002	Schrier RW, et al	Effects of aggressive blood pressure control in normotensive type 2 diabetic patients on albuminuria, retinopathy and strokes	Kidney Int	571 (30.05)	CS	BP
9	2013	Fried LF, et al.	Combined angiotensin inhibition for the treatment of diabetic nephropathy	Kidney Int	552 (69.00)	CS	RAAS
10	2004	de Zeeuw D, et al	Albuminuria, a therapeutic target for cardiovascular protection in type 2 diabetic patients with nephropathy	Circulation	544 (32.00)	CS	RAAS
11	2010	Tervaert TWC, et al	Pathologic classification of diabetic nephropathy	J Am Soc Nephrol	539 (49.00)	PP	Undefined
12	2008	Zeisberg EM, et al	Fibroblasts in kidney fibrosis emerge via endothelial-to-mesenchymal transition	J Am Soc Nephrol	533 (40.92)	MG	Fibroblast
13	2007	Kato M, et al	MicroRNA-192 in diabetic kidney glomeruli and its function in TGF-beta-induced collagen expression via inhibition of E-box repressors	Proc Natl Acad Sci U S A	518 (36.93)	MG	MiR
14	2003	Perkins BA, et al	Regression of microalbuminuria in type 1 diabetes	N Engl J Med	497 (27.61)	CS	T1DM
15	2011	Haller H, et al	Olmesartan for the delay or prevention of microalbuminuria in type 2 diabetes	N Engl J Med	494 (49.40)	CS	RAAS
16	2011	de Boer IH, et al	Temporal trends in the prevalence of diabetic kidney disease in the United States	JAMA	493 (49.30)	ES	Undefined
17	2010	de Zeeuw D, et al	Selective vitamin D receptor activation with paricalcitol for reduction of albuminuria in patients with type 2 diabetes (VITAL study): a randomised controlled trial	Lancet	482 (43.82)	CS	RAAS
18	2013	de Zeeuw D, et al	Bardoxolone methyl in type 2 diabetes and stage 4 chronic kidney disease	N Engl J Med	476 (59.50)	CS	OS
19	2003	Wendt TM, et al	RAGE drives the development of glomerulosclerosis and implicates podocyte activation in the pathogenesis of diabetic nephropathy	Am J Pathol	438 (24.33)	MG	AGEs/RAGE
20	2009	Kato M, Y, et al	TGF-beta activates Akt kinase through a microRNA-dependent amplifying circuit targeting PTEN	Nat Cell Biol	427 (35.58)	MG	MiR
21	2001	Yamamoto Y, et al	Development and prevention of advanced diabetic nephropathy in RAGE-overexpressing mice	J Clin Invest	390 (19.50)	MG	AGEs/RAGE
22	2005	Gorin Y, et al	Nox4 NAD(P)H oxidase mediates hypertrophy and fibronectin expression in the diabetic kidney	J Biol Chem	382 (23.88)	MG	OS
23	2001	De Vriese, A, et al	Antibodies against vascular endothelial growth factor improve early renal dysfunction in experimental diabetes	J Am Soc Nephrol	361 (18.05)	MG	VEGF
24	2000	Koya D, et al	Amelioration of accelerated diabetic mesangial expansion by treatment with a PKC beta inhibitor in diabetic db/db mice, a rodent model for type 2 diabetes	FASEB J	356 (16.95)	MG	OS
25	2004	Ichihara A, et al	Inhibition of diabetic nephropathy by a decoy peptide corresponding to the “handle” region for nonproteolytic activation of prorenin	J Clin Invest	352 (20.71)	MG	RAAS
26	2009	Groop PH, et al	The presence and severity of chronic kidney disease predicts all-cause mortality in type 1 diabetes	Diabetes	348 (29.00)	ES	Undefined
27	2003	Keane WF, et al	The risk of developing end-stage renal disease in patients with type 2 diabetes and nephropathy: the RENAAL Study	Kidney Int	345 (19.17)	CS	RAAS
28	1999	Cooper ME, et al	Increased renal expression of vascular endothelial growth factor (VEGF) and its receptor VEGFR-2 in experimental diabetes	Diabetes	345 (15.68)	MG	VEGF
29	2015	Kang HM, et al	Defective fatty acid oxidation in renal tubular epithelial cells has a key role in kidney fibrosis development	Nat Med	344 (57.33)	MG	Metabolism
30	2000	Tanji N, et al	Expression of advanced glycation end products and their cellular receptor RAGE in diabetic nephropathy and nondiabetic renal disease	J Am Soc Nephrol	343 (16.33)	MG	AGEs/RAGE
31	2004	Chow F, et al	Macrophages in mouse type 2 diabetic nephropathy: Correlation with diabetic state and progressive renal injury	Kidney Int	341 (20.06)	MG	Inflammation
32	2017	Mann JFE, et al	Liraglutide and renal outcomes in type 2 diabetes	N Engl J Med	337 (84.00)	CS	GLP-1
33	2011	Inoki K, et al	mTORC1 activation in podocytes is a critical step in the development of diabetic nephropathy in mice	J Clin Invest	335 (33.50)	MG	Podocyte
34	2012	Wang B, et al	Suppression of microRNA-29 expression by TGF-beta 1 promotes collagen expression and renal fibrosis	J Am Soc Nephrol	329 (36.44)	MG	MiR
35	2003	Sato A, et al	Effectiveness of aldosterone blockade in patients with diabetic nephropathy	Hypertension	326 (18.11)	CS	RAAS
36	2013	Yale JF, et al	Efficacy and safety of canagliflozin in subjects with type 2 diabetes and chronic kidney disease	Diabetes Obes Metab	324 (40.38)	CS	SGLT-2
37	2011	Godel M, et al	Role of mTOR in podocyte function and diabetic nephropathy in humans and mice	J Clin Invest	316 (31.60)	MG	Podocyte
38	2001	Oldfield MD, et al	Advanced glycation end products cause epithelial-myofibroblast transdifferentiation via the receptor for advanced glycation end products (RAGE)	J Clin Invest	316 (15.80)	MG	AGEs/RAGE
39	2002	Onozato ML, et al	Oxidative stress and nitric oxide synthase in rat diabetic nephropathy: effects of ACEI and ARB	Kidney Int	314 (16.53)	MG	RAAS
40	1999	Murphy M, et al	Suppression subtractive hybridization identifies high glucose levels as a stimulus for expression of connective tissue growth factor and other genes in human mesangial cells	J Biol Chem	313 (14.23)	MG	CTGF
41	2003	Berl T, et al	Cardiovascular outcomes in the Irbesartan Diabetic Nephropathy Trial of patients with type 2 diabetes and overt nephropathy	Ann Intern Med	306 (17.00)	CS	RAAS
42	2000	Riser BL, et al	Regulation of connective tissue growth factor activity in cultured rat mesangial cells and its expression in experimental diabetic glomerulosclerosis	J Am Soc Nephrol	305 (14.52)	MG	CTGF
43	2006	Chow FY, et al	Monocyte chemoattractant protein-1 promotes the development of diabetic renal injury in streptozotocin-treated mice	Kidney Int	304 (20.27)	MG	Inflammation
44	2004	Bolton WK, et al	Randomized trial of an inhibitor of formation of advanced glycation end products in diabetic nephropathy	Am J Nephrol	302 (17.76)	CS	AGEs/RAGE
45	2011	Zheng HT, et al	Therapeutic potential of Nrf2 activators in streptozotocin-induced diabetic nephropathy	Diabetes	302 (30.10)	MG	OS
46	2006	Parving HH, et al	Prevalence and risk factors for microalbuminuria in a referred cohort of type II diabetic patients: a global perspective	Kidney Int	299 (19.93)	ES	Undefined
47	2001	Steffes MW, et al	Glomerular cell number in normal subjects and in type 1 diabetic patients	Kidney Int	289 (14.45)	PP	Undefined
48	2008	Wang Q, et al	MicroRNA-377 is up-regulated and can lead to increased fibronectin production in diabetic nephropathy	FASEB J	288 (22.15)	MG	MiR
49	2006	Schmid H, et al	Modular activation of nuclear factor-kappa B transcriptional programs in human diabetic nephropathy	Diabetes	279 (18.60)	MG	TF
50	2010	Mann JFE, et al	Avosentan for overt diabetic nephropathy	J Am Soc Nephrol	277 (25.18)	CS	RAAS
51	2002	Yamagishi S, et al	Advanced glycation end product-induced apoptosis and overexpression of vascular endothelial growth factor and monocyte chemoattractant protein-1 in human-cultured mesangial cells	J Biol Chem	275 (14.47)	MG	AGEs/RAGE
52	2003	Bakris GL, et al	Effects of blood pressure level on progression of diabetic nephropathy - results from the RENAAL study	Arch Intern Med	274 (15.22)	CS	RAAS
53	2007	Isermann B, et al	Activated protein C protects against diabetic nephropathy by inhibiting endothelial and podocyte apoptosis	Nat Med	266 (19.00)	MG	Inflammation
54	2008	Niranjan T, et al	The Notch pathway in podocytes plays a role in the development of glomerular disease	Nat Med	264 (20.23)	MG	Podocyte
55	2007	Nakagawa T, et al	Diabetic endothelial nitric oxide synthase knockout mice develop advanced diabetic nephropathy	J Am Soc Nephrol	262 (18.71)	MG	OS
56	2003	Babaei-Jadidi R, et al	Prevention of incipient diabetic nephropathy by high-dose thiamine and benfotiamine	Diabetes	262 (14.56)	MG	Metabolism
57	2010	Krupa A, et al	Loss of MicroRNA-192 promotes fibrogenesis in diabetic nephropathy	J Am Soc Nephrol	259 (23.55)	MG	MiR
58	2005	Thorn LM, et al	Metabolic syndrome in type 1 diabetes - association with diabetic nephropathy and glycemic control (the FinnDiane study)	Diabetes Care	259 (16.19)	ES	Undefined
59	2003	Doublier S, et al	Nephrin expression is reduced in human diabetic nephropathy - evidence for a distinct role for glycated albumin and angiotensin II	Diabetes	259 (14.39)	MG	Podocyte
60	2009	Coughlan MT, et al	RAGE-induced cytosolic ROS promote mitochondrial superoxide generation in diabetes	J Am Soc Nephrol	258 (21.50)	MG	AGEs/RAGE
61	2004	Mezzano S, et al	NF-kappa B activation and overexpression of regulated genes in human diabetic nephropathy	Nephrol Dial Transplant	256 (15.06)	MG	TF
62	2005	Atkins RC, et al	Proteinuria reduction and progression to renal failure in patients with type 2 diabetes mellitus and overt nephropathy	Am J Kidney Dis	252 (15.75)	CS	RAAS
63	2005	Satoh M, et al	NAD(P)H oxidase and uncoupled nitric oxide synthase are major sources of glomerular superoxide in rats with experimental diabetic nephropathy	Am J Physiol Renal Physiol	251 (15.69)	MG	OS
64	2005	Pohl MA, et al	Independent and additive impact of blood pressure control and angiotensin II receptor blockade on renal outcomes in the Irbesartan Diabetic Nephropathy Trial: clinical implications and limitations	J Am Soc Nephrol	248 (15.50)	CS	RAAS
65	2013	Sharma K, et al	Metabolomics reveals signature of mitochondrial dysfunction in diabetic kidney disease	J Am Soc Nephrol	247 (30.88)	MG	Metabolism
66	2007	Perkins BA, et al	Microalbuminuria and the risk for early progressive renal function decline in type 1 diabetes	J Am Soc Nephrol	247 (17.64)	CS	T1DM
67	2010	Sedeek M, et al	Critical role of Nox4-based NADPH oxidase in glucose-induced oxidative stress in the kidney: implications in type 2 diabetic nephropathy	Am J Physiol Renal Physiol	246 (22.36)	MG	OS
68	2000	Wada T, et al	Up-regulation of monocyte chemoattractant protein-1 in tubulointerstitial lesions of human diabetic nephropathy	Kidney Int	246 (11.71)	MG	Inflammation
69	2011	Woroniecka KI, et al	Transcriptome analysis of human diabetic kidney disease	Diabetes	242 (24.20)	MG	TF
70	2003	Kiritoshi S, et al	Reactive oxygen species from mitochondria induce cyclooxygenase-2 gene expression in human mesangial cells - potential role in diabetic nephropathy	Diabetes	240 (13.33)	MG	OS
71	2003	Tikellis C, et al	Characterization of renal angiotensin-converting enzyme 2 in diabetic nephropathy	Hypertension	239 (13.28)	MG	RAAS
72	2012	Wang WJ, et al	Mitochondrial fission triggered by hyperglycemia Is mediated by ROCK1 activation in podocytes and endothelial cells	Cell Metab	238 (26.44)	MG	OS
73	2010	Jiang T, et al	The protective role of Nrf2 in streptozotocin-induced diabetic nephropathy	Diabetes	238 (21.55)	MG	OS
74	2002	Flyvbjerg A, et al	Amelioration of long-term renal changes in obese type 2 diabetic mice by a neutralizing vascular endothelial growth factor antibody	Diabetes	237 (12.47)	MG	VEGF
75	2007	Eijkelkamp WBA, et al	Albuminuria is a target for renoprotective therapy independent from blood pressure in patients with type 2 diabetic nephropathy: post hoc analysis from the reduction of endpoints in NIDDM with the angiotensin II antagonist losartan (RENAAL) trial	J Am Soc Nephrol	236 (16.86)	CS	RAAS
76	2003	Young BA, et al	Racial differences in diabetic nephropathy, cardiovascular disease, and mortality in a national population of veterans	Diabetes Care	236 (13.11)	ES	Undefined
77	2008	Li YJ, et al	Epithelial-to-mesenchymal transition is a potential pathway leading to podocyte dysfunction and proteinuria	Am J Pathol	235 (18.08)	MG	Podocyte
78	2011	Wang B, et al.	MiR-200a prevents renal fibrogenesis through repression of TGF-beta 2 expression	Diabetes	234 (23.40)	MG	MiR
79	2012	Niewczas MA, et al	Circulating TNF receptors 1 and 2 predict ESRD in type 2 diabetes	J Am Soc Nephrol	233 (25.89)	MG	Inflammation
80	2009	Mehdi UF, et al	Addition of angiotensin receptor blockade or mineralocorticoid antagonism to maximal angiotensin-converting enzyme inhibition in diabetic nephropathy	J Am Soc Nephrol	232 (19.33)	CS	RAAS
81	2008	Thallas-Bonke V, et al	Inhibition of NADPH oxidase prevents advanced glycation end product-mediated damage in diabetic nephropathy through a protein kinase C-alpha-dependent pathway	Diabetes	232 (17.85)	MG	OS
82	2005	Jiang T, et al	Diet-induced obesity in C57BL/6 J mice causes increased renal lipid accumulation and glomerulosclerosis via a sterol regulatory element-binding protein-1c-dependent pathway	J Biol Chem	232 (14.50)	MG	Metabolism
83	2012	Putta S, et al	Inhibiting microRNA-192 ameliorates renal fibrosis in diabetic nephropathy	J Am Soc Nephrol	231 (25.67)	MG	MiR
84	2008	Feldman DL, et al	Effects of aliskiren on blood pressure, albuminuria, and (pro)renin receptor expression in diabetic TG(mRen-2)27 rats	Hypertension	231 (17.77)	MG	RAAS
85	2006	Zhao HJ, et al	Endothelial nitric oxide synthase deficiency produces accelerated nephropathy in diabetic mice	J Am Soc Nephrol	231 (15.40)	MG	OS
86	2005	Berl T, et al	Impact of achieved blood pressure on cardiovascular outcomes in the irbesartan diabetic nephropathy trial	J Am Soc Nephrol	228 (14.25)	CS	RAAS
87	2000	Nakamura T, et al	Urinary excretion of podocytes in patients with diabetic nephropathy	Nephrol Dial Transplant	228 (10.86)	MG	Podocyte
88	2011	Palsamy P, et al	Resveratrol protects diabetic kidney by attenuating hyperglycemia-mediated oxidative stress and renal inflammatory cytokines via Nrf2-Keap1 signaling	Biochim Biophys Acta	227 (22.70)	MG	OS
89	2013	Dugan LL, et al	AMPK dysregulation promotes diabetes-related reduction of superoxide and mitochondrial function	J Clin Invest	226 (28.25)	MG	OS
90	2003	Okada S, et al	Intercellular adhesion molecule-1-deficient mice are resistant against renal injury after induction of diabetes	Diabetes	226 (12.56)	MG	Inflammation
91	2005	Asaba K, et al	Effects of NADPH oxidase inhibitor in diabetic nephropathy	Kidney Int	225 (14.06)	MG	OS
92	2002	Forbes JM, et al	Reduction of the accumulation of advanced glycation end products by ACE inhibition in experimental diabetic nephropathy	Diabetes	223 (11.74)	MG	RAAS
93	2015	Bakris GL, et al	Effect of finerenone on albuminuria in patients with diabetic nephropathy: a randomized clinical trial	JAMA	221 (36.83)	CS	RAAS
94	2002	Ha HJ, et al	Role of high glucose-induced nuclear factor-kappa B activation in monocyte chemoattractant protein-1 expression by mesangial cells	J Am Soc Nephrol	221 (11.63)	MG	TF
95	2006	Ichihara A, et al	Prorenin receptor blockade inhibits development of glomerulosclerosis in diabetic angiotensin II type 1a receptor-deficient mice	J Am Soc Nephrol	220 (14.67)	MG	RAAS
96	2014	Vallon V, et al	SGLT2 inhibitor empagliflozin reduces renal growth and albuminuria in proportion to hyperglycemia and prevents glomerular hyperfiltration in diabetic Akita mice	Am J Physiol Renal Physiol	219 (31.29)	MG	SGLT-2
97	2008	Zhang Z, et al	Combination therapy with AT1 blocker and vitamin D analog markedly ameliorates diabetic nephropathy: blockade of compensatory renin increase	Proc Natl Acad Sci U S A	218 (16.77)	MG	RAAS
98	2016	Afkarian M, et al	Clinical manifestations of kidney disease among US adults with diabetes, 1988–2014	JAMA	217 (43.40)	ES	Undefined
99	2013	Zhong X, et al	MiR-21 is a key therapeutic target for renal injury in a mouse model of type 2 diabetes	Diabetologia	217 (27.13)	MG	MiR
100	2011	Kitada M, et al	Resveratrol improves oxidative stress and protects against diabetic nephropathy through normalization of Mn-SOD dysfunction in AMPK/SIRT1-independent pathway	Diabetes	217 (27.13)	MG	OS

### Distributions of the top 100 most cited articles in regard to different subcategories, topics and periods

**Fig. 4:**
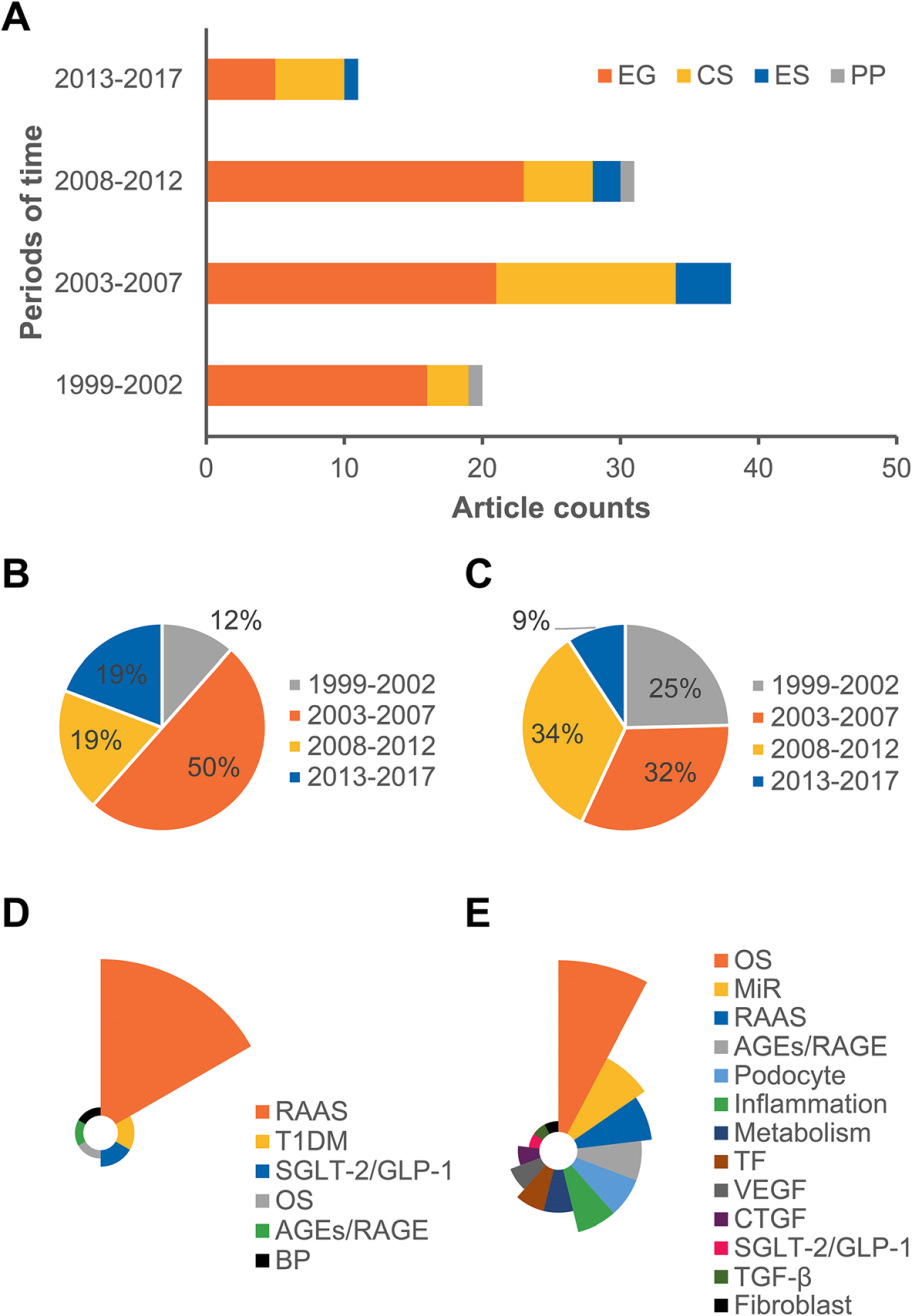
Distributions of the subcategories, topics and time periods. The distributions of the 100 articles are shown in the figure (**a**). The distributions of clinical studies (**b**) and experimental studies (**c**) in different time periods are shown as pie charts. The distributions of topics in clinical (**d**) and experimental studies (**e**) are showed as Nightingale rose charts

In Fig. 4, we show the distribution of the top 100 most cited articles. We divided the year of publication into 4 periods. The distributions of the 100 articles are shown in Fig. 4a. Most ‘CS’ articles were published from 2003 to 2007 (Fig. 4b), while most ‘MG’ articles were published from 2008 to 2012 (Fig. 4c).

Intriguingly, we revealed popular research fields via topic analysis. We found that the renin-angiotensin-aldosterone system (RAAS) was the most popular topic. Clinical trials on the application of RAAS blockade in DKD (article number 1, 2, 3, 7, 9, 10, 15, 27, 41, 52, 62, 64, 75, and 86, a total of 11,852 citations, 30.50% of the total citations) included 6 of the top 10 most cited articles, which contributed to 26.52% of the total citations. Correspondingly, 6 articles (article number 25, 39, 71, 84, 92, and 95, a total of 1579 citations, 4.06% of the total citations) explained the mechanism of RAAS in DKD. In addition, RAAS blockade-based combination treatment was also popular among researchers, as it was associated with 6 articles that were cited 1756 times (4.52% of the total citations). RAAS blockade combined with vitamin D analogues (article number 17, 97) and mineralocorticoid antagonism (article number 35, 80, 93) were recommended for renal function protection, but RAAS combined with the endothelin antagonist avosentan (article number 50) was considered to induce fluid overload. Taken together, these data indicated the crucial role of RAAS blockade in therapeutic strategies for DKD.

The next most important topic was how the OS participates in DKD, which was associated with 17 articles published from 2000 to 2013 (article number 5, 18, 22, 24, 45, 55, 63, 67, 70, 72, 73, 81, 85, 88, 89, 91, and 100, a total of 5080 citations, 13.07% of the total citations). In experimental DKD, the increase in reactive oxygen species (ROS) (article number 5) and NAD(P)H oxidase levels (article number 22, 63, 67, and 91), activation of PKC (article number 24 and 81), decrease in eNOS (article number 55, 85, and 100) and mitochondrial dysfunction (article number 70, 72, and 89) promote OS injury, while Nrf2 (article number 45, 73, and 88) protects the kidney from OS in DKD as an antioxidant factor. However, a phase 3 clinical study (article number 18) found that the Nrf2 activator bardoxolone methyl did not ameliorate the loss of renal function. Instead, bardoxolone methyl led to a higher cardiovascular risk.

The accumulation of advanced glycation end products (AGEs) is also a classic pathogenic mechanism in DKD. Among the top 100 most cited articles, 7 articles (article number 19, 21, 30, 38, 44, 51, and 60, a total of 2322 citations) focused on AGEs and the receptor for AGEs (RAGE), which constituted 5.98% of the total citations. Although they slowed the progression of experimental DKD, agents targeting AGEs/RAGE were not easily translated into the clinic. In a randomized clinical trial, pimagedine, an inhibitor of AGE formation, did not ameliorate nephropathy in patients with type 1 diabetic mellitus (T1DM) (article number 44).

In addition to therapies developed based on classic mechanisms, researchers have highlighted new drugs developed based on new mechanisms in recent years. The sodium-glucose cotransporter 2 (SGLT-2) inhibitor empagliflozin and glucagon-like peptide 1 (GLP-1) agonist liraglutide are two rising stars in DKD treatment. There were 2 articles on empagliflozin among the top 100 most cited articles, one clinical trial (article number 36, 324 citations) published in 2013 and one experimental study (article number 96, 219 citations) published in 2014. Moreover, the clinical trial of liraglutide published in 2017 (article number 32, 337 citations) was the latest among the 100 articles. These new drugs represent new trends in DKD research.

Some experimental studies uncovered the crucial mechanisms of DKD, but the clinical translations of these studies were not included in the top 100 most cited articles. Fifteen articles elucidated the important roles of podocyte dysfunction (article number 33, 37, 54, 59, 77, and 87, a total of 1637 citations), immune cells and inflammation (article number 31, 43, 53, 68, 79, and 90, a total of 1616 citations), and vascular endothelial growth factor (VEGF) (article number 23, 28, 74, totally cited 943 times) in DKD, and 4 articles that were cited 1858 times demonstrated that transforming growth factor (TGF)-β (article number 6), fibroblasts (article number 12), and connective tissue growth factor (CTGF) (article number 40, 42) contribute to renal fibrosis in DKD. A new mechanism drawing the attention of researchers was the role of microRNAs (miRs) in DKD, which was discussed in 8 articles published from 2007 to 2013 (article number 13, 20, 34, 48, 57, 78, 83, and 99, a total of 2503 citations). Furthermore, metabolomics and transcriptomics analyses deepened researchers’ understanding of DKD. Targeting metabolic alterations (article number 29, 56, 65, and 82, a total of 1085 citations) and novel transcription factors (TFs) (article number 49, 61, 69, and 94, a total of 998 citations) had therapeutic effects in experimental DKD.

The articles above revealed the mechanism of and treatment strategies for DKD. In the remaining clinical studies, one article (article number 8, 571 citations) emphasized the importance of blood pressure (BP) control in DKD, and 2 articles discussed microalbuminuria (article number 14, 66, a total of 744 citations) in T1DM. Figure 4d and e show the distributions of topics in ‘CS’ and ‘MG’ articles respectively. Furthermore, in Table 5, we separately list the information and results of clinical studies in regard to their critical roles in clinical guidance.

**Table 5:** List of the clinical studies among the top 100 most cited articles

Information	Topic	Patients	Main results
**Article number 1:** Effects of losartan on renal and cardiovascular outcomes in patients with type 2 diabetes and nephropathy **ACT:** 234.95	RAAS	T2DM patients with nephropathy	Losartan reduced the incidence of a doubling of the serum creatinine concentration, the incidence of end-stage renal disease, and the level of proteinuria by 25, 28, and 35%, respectively. In addition, losartan did not significantly alter the morbidity and mortality from cardiovascular causes.
**Article number 2:** The effect of irbesartan on the development of diabetic nephropathy in patients with type 2 diabetes **ACT:** 111.45	RAAS	Hypertensive patients with T2DM and microalbuminuria	Irbesartan reduced the incidence of nephropathy and the level of urinary albumin excretion independently of its antihypertensive effect. In addition, irbesartan also reduced the incidence of serious adverse events.
**Article number 3:** Aliskiren combined with losartan in type 2 diabetes and nephropathy **ACT:** 59.15	RAAS	Hypertensive patients with T2DM and nephropathy	Aliskiren reduced the mean UACR by 20% without significant change in the total numbers of adverse and serious adverse events.
**Article number 7:** Proteinuria, a target for renoprotection in patients with type 2 diabetic nephropathy: lessons from RENAAL **ACT:** 39.76	RAAS	T2DM patients with nephropathy	Baseline albuminuria was the predominant renal risk parameter. The residual albuminuria after 6 months of losartan treatment was another strong renal risk parameter. Losartan reduced the level of albuminuria, which was the major explanation of its renoprotective function.
**Article number 8:** Effects of aggressive blood pressure control in normotensive type 2 diabetic patients on albuminuria, retinopathy and strokes **ACT:** 30.05	BP	Normotensive T2DM patients	Over a 5-year follow-up period, intensive BP control (approximately 128/75 mmHg) reduced the percentage of normoalbuminuria-to-microalbuminuria and microalbuminuria-to-overt albuminuria, the progression of diabetic retinopathy and the incidence of stroke compared to the moderate BP control group (approximately 137/81 mmHg). In addition, intensive BP control had no significant influence on creatinine clearance.
**Article number 9:** Combined angiotensin inhibition for the treatment of diabetic nephropathy **ACT:** 69.00	RAAS	T2DM veterans with overt nephropathy	Over a 2-year follow-up period, the combination of ACEi and ARB induced hyperkalemia and acute kidney injury. For safety concerns, the study was stopped.
**Article number 10:** Albuminuria, a therapeutic target for cardiovascular protection in type 2 diabetic patients with nephropathy **ACT:** 32.00	RAAS	T2DM patients with nephropathy	Baseline albuminuria was the predominant risk predictor of cardiovascular outcome. Reducing albuminuria in the first 6 months was renoprotective.
**Article number 14:** Regression of microalbuminuria in type 1 diabetes **ACT:** 27.61	T1DM	T1DM patients with microalbuminuria	Over a 6-year follow-up period, the incidence of regression of microalbuminuria was 58%. The regression of microalbuminuria did not indicate inexorable progression of nephropathy, and ACEi treatment was not associated with the regression of microalbuminuria.
**Article number 15:** Olmesartan for the delay or prevention of microalbuminuria in type 2 diabetes **ACT:** 49.40	RAAS	T2DM patients	Over a 3-year follow-up period, olmesartan delayed the onset of microalbuminuria by 23%. Notably, olmesartan increased the risk of fatal cardiovascular events among T2DM patients with preexisting coronary heart disease.
**Article number 17:** Selective vitamin D receptor activation with paricalcitol for reduction of albuminuria in patients with type 2 diabetes (VITAL study): a randomised controlled trial **ACT:** 43.82	RAAS	T2DM patients with albuminuria and ACEi or ARB treatment	Addition of 2 μg/day paricalcitol to RAAS inhibition safely reduced UACR by 18 to 28% compared with placebo.
**Article number 18:** Bardoxolone methyl in type 2 diabetes and stage 4 chronic kidney disease **ACT:** 59.50	OS	T2DM patients with stage 4 chronic kidney disease	Over a 9-month follow-up period, bardoxolone methyl did not reduce the risk of end-stage renal disease or death from cardiovascular causes but induced cardiovascular events. For safety concerns, the study was stopped.
**Article number 27:** The risk of developing end-stage renal disease in patients with type 2 diabetes and nephropathy: The RENAAL Study **ACT:** 19.17	RAAS	T2DM patients with nephropathy	A multivariate model showed that proteinuria, serum creatinine, serum albumin and haemoglobin level were independent risk predictors of renal outcomes after control of BP. Moreover, the level of proteinuria was the most important risk parameter for progressive kidney injury.
**Article number 32:** Liraglutide and renal outcomes in type 2 diabetes **ACT:** 84.00	GLP-1	T2DM patients with high risk for cardiovascular disease	Over a 3-year follow-up period, liraglutide reduced the new onset of persistent macroalbuminuria by 26% and the risk of the development and progression of DKD.
**Article number 35:** Effectiveness of aldosterone blockade in patients with diabetic nephropathy **ACT:** 18.11	RAAS	T2DM patients with early nephropathy and ACEi treatment	Aldosterone escape appeared in 40% of patients and led to less effective antiproteinuric effects of ACEi treatment. Over a 6-month follow-up period, addition of spironolactone to ACEi reduced UACR and left ventricular mass index while it did not significantly influence BP.
**Article number 36:** Efficacy and safety of canagliflozin in subjects with type 2 diabetes and chronic kidney disease **ACT:** 40.38	SGLT-2	T2DM patients with stage 3 chronic kidney disease	Canagliflozin reduced HbA1c and fasting plasma glucose and was generally well tolerated.
**Article number 41:** Cardiovascular outcomes in the Irbesartan Diabetic Nephropathy Trial of patients with type 2 diabetes and overt nephropathy **ACT:** 17.00	RAAS	T2DM patients with overt nephropathy	Irbesartan or amlodipine in addition to conventional antihypertensive therapy did not significantly influence the risk of composite cardiovascular events compared to placebo in addition to conventional antihypertensive therapy.
**Article number 44:** Randomized trial of an inhibitor of formation of advanced glycation end products in diabetic nephropathy **ACT:** 17.76	AGEs/RAGE	T1DM patients with nephropathy and retinopathy	Pimagedine did not significantly influence the progression of overt nephropathy.
**Article number 50:** Avosentan for overt diabetic nephropathy **ACT:** 25.18	OS	T2DM patients with ACEi or ARB treatment	Over a 4-month follow-up period, addition of avosentan to RAAS inhibitor reduced albuminuria but induced significant fluid overload and congestive heart failure. For safety concerns, the study was stopped.
**Article number 52:** Effects of blood pressure level on progression of diabetic nephropathy - results from the RENAAL study **ACT:** 15.22	RAAS	T2DM patients with nephropathy and hypertension	Baseline systolic blood pressure was a stronger risk predictor than diastolic blood pressure of renal outcomes. Patients with the highest baseline pulse pressure had the highest renal risk and benefited most after losartan-reduced systolic blood pressure.
**Article number 62:** Proteinuria reduction and progression to renal failure in patients with type 2 diabetes mellitus and overt nephropathy **ACT:** 15.75	RAAS	T2DM patients with overt nephropathy	Each doubling of baseline proteinuria level doubled the risk for renal failure. Each halving of proteinuria level between baseline and 12 months with treatment reduced the risk for renal failure by 56%. With the same reduction in proteinuria, irbesartan reduced more risk of renal failure compared to amlodipine.
**Article number 64:** Independent and additive impact of blood pressure control and angiotensin II receptor blockade on renal outcomes in the Irbesartan Diabetic Nephropathy Trial: clinical implications and limitations **ACT:** 15.50	RAAS	Hypertensive T2DM patients with overt nephropathy	Systolic blood pressure was the strong risk predictor of baseline serum creatinine doubling or end-stage renal disease. Systolic blood pressure target between 120 and 130 mmHg combined with irbesartan was recommended. There was no correlation between diastolic blood pressure and renal outcomes.
**Article number 66:** Microalbuminuria and the risk for early progressive renal function decline in type 1 diabetes **ACT:** 17.64	T1DM	T1DM patients	Early renal function decline occurred in 9% of the normoalbuminuria group and 31% of the microalbuminuria group. In multivariate analysis, the risk of early renal function decline increased after 35 years old or HbA1c exceeding 9% but was not influenced by diabetes duration, smoking, BP or ACEi treatment. Cystatin C together with microalbuminuria was recommended to diagnose early renal function decline.
**Article number 75:** Albuminuria is a target for renoprotective therapy independent from blood pressure in patients with type 2 diabetic nephropathy: post hoc analysis from the reduction of endpoints in NIDDM with the angiotensin II antagonist losartan (RENAAL) trial **ACT:** 16.86	RAAS	T2DM patients with hypertension	Systolic blood pressure reduction, albuminuria regression and low level of residual albuminuria were associated with a lower risk of end-stage renal disease. Systolic blood pressure reduction together with albuminuria regression was recommended to be the target of antihypertensive treatment to improve renal outcome.
**Article number 80:** Addition of angiotensin receptor blockade or mineralocorticoid antagonism to maximal angiotensin-converting enzyme inhibition in diabetic nephropathy **ACT:** 19.33	RAAS	Hypertensive T2DM patients with albuminuria	Compared to lisinopril alone, addition of spironolactone to lisinopril reduced UACR by 36% and addition of losartan to lisinopril reduced UACR by 16.8%. Spironolactone was recommended to be combined with lisinopril for a greater renoprotective function in addition to BP control.
**Article number 86:** Impact of achieved blood pressure on cardiovascular outcomes in the irbesartan diabetic nephropathy trial **ACT:** 14.25	RAAS	T2DM patients with overt nephropathy	Progressively lower achieved BP 120/85 mmHg was associated with the best protection against cardiovascular events. Increased pulse pressure, systolic blood pressure below 120 mmHg or diastolic blood pressure below 85 mmHg was associated with increased cardiovascular events.
**Article number 93:** Effect of finerenone on albuminuria in patients with diabetic nephropathy: a randomized clinical trial **ACT:** 36.83	RAAS	Diabetic patients with high or very high albuminuria and ACEi or ARB treatment	Addition of finerenone to ACEi or ARB dose-dependently reduced UACR and was well tolerated.

Regarding other article types, 9 articles subcategorized as ‘ES’ and ‘PP’ helped researchers understand DKD from disparate aspects. Seven articles (article number 4, 16, 26, 46, 58, 76, and 99, a total of 2603 citations) were based on the epidemiological aspects of DKD, and 2 articles (article number 11, 47, a total of 828 citations) defined the pathological characteristics and classifications of DKD. Four of the 9 articles (article number 4, 26, 47, and 58) were specifically on nephropathy in T1DM.

### Evolution of topics in clinical and experimental studies of DKD

To elucidate the trends in DKD research, we analysed the distributions of topics of ‘CS’ and ‘MG’ articles in different periods and illustrated their evolution, as shown in Figure S2. Interestingly, we found that RAAS and OS are continuous hotspots of DKD research, again emphasizing the significance of the two research fields. Although attention has been given in the past, the number of studies on AGEs and RAGE has declined in recent years, which may be due to the frustration of clinical translation. Articles on miRs were predominately concentrated from 2008 to 2012, indicating the trendy miR-related study outbreak and attraction of numerous researchers in those years. In addition, some topics have appeared in recent years, including SGLT-2 and GLP-1, which have become new hotspots and have led to novel breakthroughs in DKD studies.

## Discussion

In this study, we ranked the top 100 most cited articles on DKD according to the total number of citations in the Web of Science Core Collection. In addition, we analysed the journals, timespans, authors, countries and topics of the 100 articles. This bibliometric analysis helps readers quickly understand the influential studies in DKD research, which topics attract other researchers, and the evolution of the research trend, thus guiding researchers to find interesting research directions and may help facilitate international collaborations. Popular clinical studies guide readers in clinical practice to provide more benefits for patients. Important experimental studies provide laboratory evidence for clinical trials and deepen the comprehension of the development and progression of DKD. Although some studies have not yet been clinically translated, they may direct future research and provide potential biomarkers and therapeutic targets as the subject develops in the future.

Bibliometric analysis is an effective method that is utilized in diverse areas of study [12–16, 18–20]. A bibliometric analysis performed in 2019 [19] showed that 3 of the top 10 most cited articles in nephrology were on the subject of RAAS blockade usage in DKD. A former bibliometric analysis of DKD [20] highlighted the authors and co-citation networks but not the subcategories or contents. In this study, we further performed a bibliometric analysis of the top 100 most cited articles to elucidate what has been done and what needs to be completed in DKD research. In addition, there were some limitations in this study. We performed this study based on data from the Web of Science Core Collection, which means that some high-quality articles that were published earlier or were not in English were potentially excluded from this study. Another limitation is that the drawback of the bibliometric citation analysis method may contain bias, as recent articles have less time to be cited. Thus, we also listed the number of citations for each article per year, which helps to highlight the influence of recent studies [21] in addition to the total number of citations. On the other hand, we discussed the recent advances in traditional topics and more findings in the latest hotspots.

The most relevant field and a persistent interest in DKD research is RAAS, which was associated with most articles and citations. In the latest Kidney Disease: Improving Global Outcomes guidelines [1], angiotensin-converting enzyme inhibitors (ACEis) and angiotensin-II receptor blockers (ARBs) are recommended for use in DKD patients. In addition to ACEis and ARBs, there are other types of RAAS blockades, including direct renin inhibitors and ectoenzyme neutral endopeptidase inhibitors. In 2008, a randomized study showed that the combination of losartan and aliskiren, a direct renin inhibitor, significantly reduced the mean urine albumin-to-creatinine ratio (UACR) compared with that achieved with losartan combined with placebo in T2DM patients (article number 3, 751 citations). In 2018, the intention-to-treat analysis of UK HARP-III [22] showed that sacubitril, an ectoenzyme neutral endopeptidase inhibitor, combined with valsartan had a similar renal benefit and additional heart function protection when compared to that achieved with irbesartan in patients with an estimated glomerular filtration rate of 60 to 20 mL/min/1.73 m^2^. The intention-to-treat analysis of PARADIGM-HF [23] showed that sacubitril combined with valsartan took advantage of delaying renal function decline and protecting heart function when compared with enalapril in patients with diabetes. Novel RAAS inhibitors have promising prospects in the treatment of DKD owing to their advantages.

Notably, article number 71 (239 citations) highlights the expression of angiotensin-converting enzyme 2 (ACE2) in DKD. However, the expression of ACE2 in DKD is controversial [24, 25]. One recent study [26] found that the mRNA level of ACE2 was increased in the proximal tubular epithelial cells of DKD patients. In addition, some studies [27, 28] have shown that RAAS inhibitors upregulate the expression of ACE2. ACE2 is the receptor for SARS-CoV-2 [29–31], which is responsible for the COVID-19 outbreak, giving rise to the following two questions: will renal injury be increased in COVID-19 patients with diabetes and will ACEi/ARB treatment have an impact on the renal outcome of COVID-19 patients with diabetes? COVID-19 patients with T2DM have higher acute kidney injury prevalence rates than individuals without diabetes [32], which may be explained by ACE2-mediated viral cytopathic effects [26, 33]. Some studies [34, 35] implied that RAAS inhibitors increased the risk of acute kidney injury, suggesting more concern about renal outcomes in patients with severe COVID-19 with diabetes who are treated with ACEis/ARBs. More studies are needed to further elucidate the roles of ACE2 and ACEi/ARB-induced ACE2 in DKD.

Strategies for protecting renal function are a permanent topic of DKD. Although RAAS blockade benefits a substantial population of patients, there are still some questions regarding DKD treatment. Unfortunately, the development of novel drugs based on new mechanisms in DKD treatment has been uneven. Fortunately, two novel types of drugs, SGLT-2 inhibitors and GLP-1 receptor agonists, may represent the future of DKD treatment methods. CREDENCE [36] and the following analysis of CREDENCE [37, 38] revealed that canagliflozin reduces the risk of renal failure and cardiovascular events and decreases anaemia-associated outcomes in T2DM patients with kidney disease. DAPA-CKD [39] showed that dapagliflozin, another SGLT-2 inhibitor, reduces kidney and cardiovascular events in chronic kidney disease patients with and without T2DM. Intriguingly, the latest publication among the top 100 most cited articles is a clinical trial of another new drug, liraglutide, a GLP-1 receptor agonists (article number 32), which has been proven to reduce the occurrence of persistent macroalbuminuria. Encouragingly, the two novel types of agents showed more advantages for kidney function protection than dipeptidyl peptidase-4 inhibitor or sulfonylureas [40] and were recommended by the Kidney Disease: Improving Global Outcomes guidelines guidelines [1] and consensus report by American Diabetes Association and the European Association for the study of diabetes [41, 42] in T2DM patients with chronic kidney disease. To develop more novel targets of DKD treatment, further comprehension of the mechanisms of DKD is indispensable.

Technological innovation promotes scientific development. High-throughput technology and multiomics studies help researchers further understand the pathogenesis and prognosis of DKD (article number 65, 69). The application of single-cell and single-nucleus transcriptomics [43–45] identifies novel types of cells, describes cell-to-cell crosstalk, discovers further mechanisms and provides new insight into renal diseases. Further understanding the disease will help researchers find potential biomarkers and novel therapeutic strategies in the future.

Moreover, artificial intelligence is widely applied in the analysis of clinical indicators, digital imaging data, and digital pathological data in renal diseases [46, 47] and improves the diagnosis and prognostication of DKD [48–50]. High-performance models built by artificial intelligence may contribute to more effective and accurate interventions in the clinical practice of DKD.

## Conclusion

In conclusion, this article focused on the top 100 most cited articles on the subject of DKD. By reviewing the popular studies over several decades, researchers can better understand the evolution of DKD research. The important roles of ACEis and ARBs were once again emphasized in this study, and the prospects of SGLT-2 inhibitors and GLP-1 receptor activators are promising. Influential studies deepen the understanding of DKD and provide evidence for novel biomarkers and potential therapeutic strategies. This study helps readers quickly understand the important studies on DKD research, the distribution of popular topics and the evolution of the subject, thus providing a guide for research direction, international collaboration and clinical practice to better server patients.

## Supplementary Information

**Additional file 1: Figure S1.** The top 10 relevant authors who contributed to the top 100 most cited articles. The dot size represents the author’s score, and the dot colour reflets the number of articles. Black represents one article, blue represents two articles, yellow represents three articles, and orange represents four articles. The authors’ contributions are exhibited as a bubble plot.

**Additional file 2: Figure S2.** The evolution of topics in DKD research. The distributions of the topics RAAS (A), OS (B), AGEs and RAGE (C), miR (D), podocyte (E), inflammation (F), metabolism (G), TF (H), SGLT-2 and GLP-1 (I), VEGF (J), T1DM (K), CTCF (L), TGF-β (M), fibroblast (N) and BP (O) in different time periods reflect the evolution of topics in DKD research.

**Additional file 3: Table S1.** Recent reviews on the subject of DKD.

## Data Availability

All data generated or analysed during this study are included in this published article [and its supplementary information files].

## Abbreviations

DM: Diabetic mellitus
DKD: Diabetic kidney disease
CKD: Chronic kidney disease
OS: Oxidative stress
CT: Citation time
ACT: Average citation time
EG: Experimental studies
CS: Clinical studies
ES: Epidemiological studies
PP: Pathological and pathophysiological studies
RAAS: Renin-angiotensin-aldosterone system
ROS: Reactive oxygen species
AGEs: Advanced glycation end products
RAGE: Receptor for advanced glycation end products
T1DM: Type 1 diabetic mellitus
SGLT-2: Sodium-glucose cotransporter 2
GLP-1: Glucagon-like peptide 1
VEGF: Vascular endothelial growth factor
TGF: Transforming growth factor
CTGF: Connective tissue growth factor
miR: MicroRNA
TF: Transcriptional factor
BP: Blood pressure
T2DM: Type 2 diabetic mellitus
ACEi: Angiotensin-converting enzyme inhibitors
ARB: Angiotensin-II receptor blockers
UACR: Urine albumin-to-creatinine ratio
ACE2: Angiotensin-converting enzyme 2
