# Supplementary figures and images for: The evolution and future of diabetic kidney disease research: a bibliometric analysis
Source: BMC Nephrol. 2021 Apr 29;22:158. doi: 10.1186/s12882-021-02369-z (PMC8084262; doi:10.1186/s12882-021-02369-z)

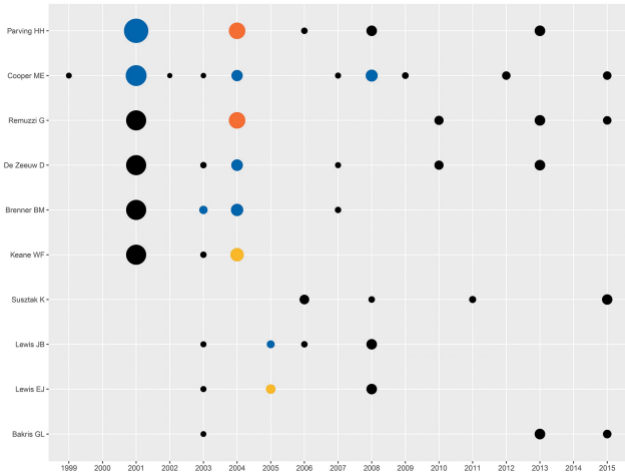

Supplement: Supplementary file 1 — Additional file 1: Figure S1. The top 10 relevant authors who contributed to the top 100 most cited articles. The dot size represents the author’s score, and the dot colour reflets the number of articles. Black represents one article, blue represents two articles, yellow represents three articles, and orange represents four articles. The authors’ contributions are exhibited as a bubble plot. [file 12882_2021_2369_MOESM1_ESM.pdf]

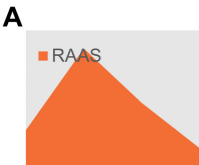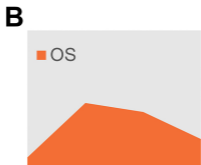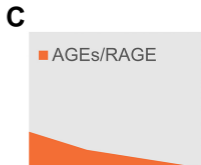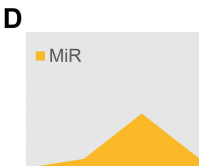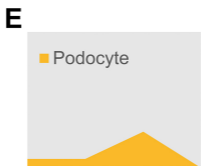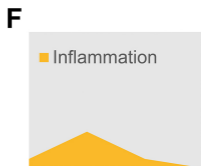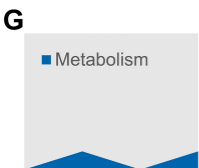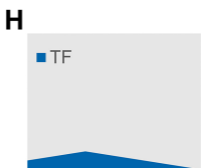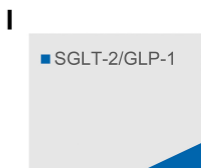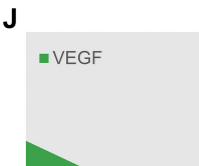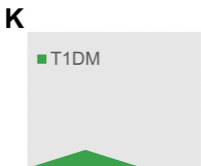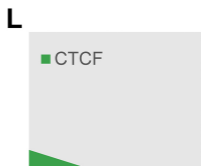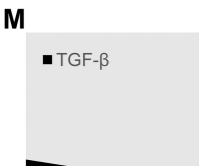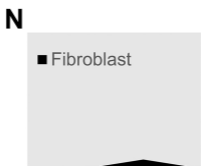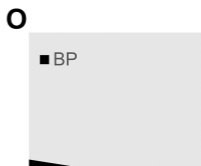

Supplement: Supplementary file 2 — Additional file 2: Figure S2. The evolution of topics in DKD research. The distributions of the topics RAAS (A), OS (B), AGEs and RAGE (C), miR (D), podocyte (E), inflammation (F), metabolism (G), TF (H), SGLT-2 and GLP-1 (I), VEGF (J), T1DM (K), CTCF (L), TGF-β (M), fibroblast (N) and BP (O) in different time periods reflect the evolution of topics in DKD research. [file 12882_2021_2369_MOESM2_ESM.pdf]
