# Supplementary material for: The evolution and future of diabetic kidney disease research: a bibliometric analysis
Source: BMC Nephrol. 2021 Apr 29;22:158. doi: 10.1186/s12882-021-02369-z (PMC8084262; doi:10.1186/s12882-021-02369-z)
Supplement: Supplementary file 3 — Additional file 3: Table S1. Recent reviews on the subject of DKD. [file 12882_2021_2369_MOESM3_ESM.docx]

Table S1. Recent reviews on the subject of DKD.

| Year | Author | Title | Journal | Topic and conclusion |
| --- | --- | --- | --- | --- |
| 2020 | Vallon V, et al[11] | The tubular hypothesis of nephron filtration and diabetic kidney disease | Nat Rev Nephrol | This review provided a tubule-centric view. Early diabetes induced the upregulation of SGLT-1 and SGLT-2 in renal tubules, which altered the interactions between the tubule and glomerulus and contributed to hyperfiltration. |
| 2020 | Donate-Correa J, et al[3] | Inflammatory targets in diabetic nephropathy | J Clin Med | This review summarized how accumulated inflammatory cells, chemokines, cytokines and inflammation related signalling pathways participate in DKD. Moreover, the anti-inflammatory effects of DKD therapies were reviewed. |
| 2020 | Tang SCW, et al[4] | Innate immunity in diabetic kidney disease | Nat Rev Nephrol | This review introduced the role of innate immunity in DKD. Toll-like receptors, inflammasomes, kallikrein–kinin system, and proteinase-activated receptors, and complement cascade orchestrated in the pathogenesis and progression of DKD. Targeting the innate immune-related pathways provided a promising therapeutic aspect. |
| 2019 | Warren AM, et al[9] | Diabetic nephropathy: an insight into molecular mechanisms and emerging therapies | Expert Opin Ther Targets | This review summarized therapies that affected haemodynamic and metabolic pathways. Understanding the DKD pathophysiology would help in the development of drugs. |
| 2019 | Kato M, et al[10] | Epigenetics and epigenomics in diabetic kidney disease and metabolic memory | Nat Rev Nephrol | This review highlighted the role of epigenetic mechanisms involving chromatin histone modifications, DNA methylation and non-coding RNAs in DKD. Epigenetic molecules were potential diagnostic biomarkers and valuable therapeutic targets. |
| 2019 | Yaribeygi H, et al[5] | Effects of antidiabetic drugs on NLRP3 inflammasome activity, with a focus on diabetic kidneys | Drug Discov Today | This review introduced that NLRP3 inflammasome participates in DKD development. Antidiabetic drugs prevented the development and progression of DKD via NLRP3 inflammasome modification. |
| 2018 | Forbes JM, et al[6] | Mitochondrial dysfunction in diabetic kidney disease | Nat Rev Nephrol | This review provided a mitochondrial-centric view. Mitochondrial dysfunction, metabolic switch and inherited factors of mitochondrial function contributed to DKD development and progression. Several promising agents that targeted mitochondrial function were under investigation in clinical trials. |

Table S1. Continued.

| Year | Author | Title | Journal | Topic and conclusion |
| --- | --- | --- | --- | --- |
| 2018 | Sifuentes-Franco S, et al[7] | Oxidative stress, apoptosis, and mitochondrial function in diabetic nephropathy | Int J Endocrinol | This review introduced OS mechanism, ROS and OS-induced apoptosis in the pathogenesis and progression of DKD. Current and potential therapies that had antioxidant effect were summarized. |
| 2018 | Sagoo MK, et al[8] | Diabetic nephropathy: Is there a role for oxidative stress? | Free Radic Biol Med | This review discussed the contradicting role of OS in DKD. Experimental studies showed that OS promotes DKD. Unfortunately, the clinical translation was disappointing. Both excessive production and inhibition of OS resulted in the development of DKD. |
